# Supplementary material for: Lyophilization induces physicochemical alterations in cryptococcal exopolysaccharide
Source: Carbohydr Polym. Author manuscript; Available in PMC 2023 Mar 31. (PMC10064552; doi:10.1016/j.carbpol.2022.119547)
Supplement: Supplemental [file NIHMS1880288-supplement-Supplemental.docx]

**Supplemental Figure 1. Solution ^1^H NMR variation observed between biological replicates of H99 EPS.** Unprocessed *C. neoformans*H99 EPS samples (Native) from two different biological replicates were examined by 1D ^1^H NMR. The sample in red is portrayed throughout this work because the peak set in the SRG region was easier to define than in replicate 2. The same reduction in signal was observed for both samples after lyophilization.


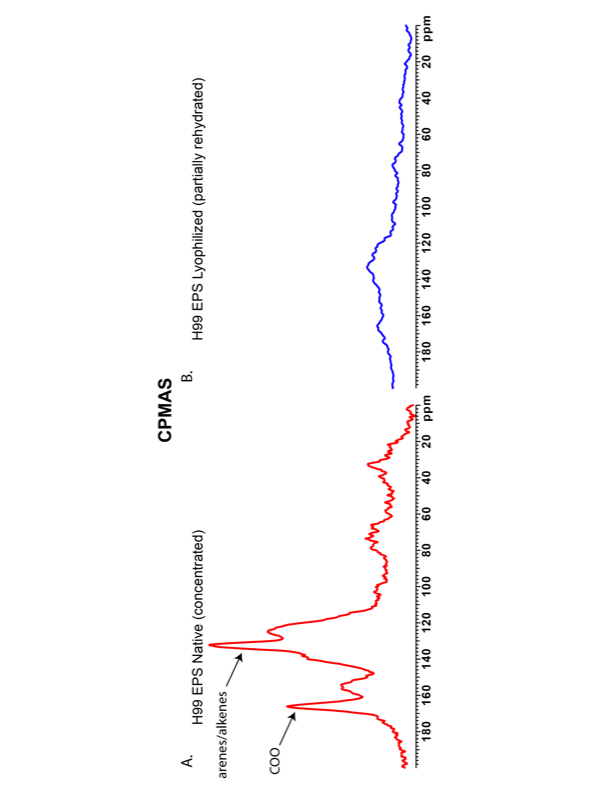


**Supplemental Figure 2. Effects of lyophilization on solid-state 13C NMR spectra of EPS.**150 MHz 13C NMR spectra of *C. neoformans*EPS samples obtained with 15 kHz magic-angle spinning (MAS). CPMAS experiments that favor rigid carbon moieties for which cross polarization from nearby hydrogen nuclei is efficient. A. Native H99 EPS (concentrated, partially dehydrated). B. Lyophilized H99 EPS (partially rehydrated).
